# Worldwide Innovative Network Consortium: Building a Common Global Cancer Database

**DOI:** 10.1200/GO-25-00720

**Published:** 2026-08-13

**Authors:** Farhood Farahnak, Wafik S. El-Deiry, Yves A. Lussier, Razelle Kurzrock, Shai Magidi, Catherine Bresson, Shirin A. Enger, Jia Liu, Jair Bar, Jeremy L. Warner, Tobias Meissner, Eitan Rubin, Jens Rueter, Himabindu Gaddipati, Mandar Kulkarni, Zhen Chen, Sewanti Limaye, Rachel Elsey, Brenda M. Rubenstein, Anthony M. Joshua, Humaid O. Al-Shamsi, Khaled M. Musallam, Fanny Wunder, Jacques Raynaud, Guy Berchem, Manon Gantenbein, Amal Al Omari, Said Dermime, Hikmat Abdel-Razeq, Pierre Saintigny, Andrés Cervantes, Roger R. Reddel, Adel T. Aref, Juan Martin-Liberal, Conxi Lázaro, David Cordero Romera, Marina Sekacheva, Raanan Berger, C.S. Pramesh, Ioana Berindan-Neagoe, Eugenia Girda, Alejandro Piris-Gimenez, Carol J. Farhangfar, Mohammed E. Salem, Rodrigo Dienstmann, Ramon Salazar, Naftali Z. Frankel, Zachary Batist, Yuri Quintana, Gerald Batist

**Affiliations:** ^1^ McGill University Centre for Translational Research in Cancer and Segal Cancer Centre, Jewish General Hospital, Montreal, QC, Canada; ^2^ Worldwide Innovative Network (WIN) Association—WIN Consortium, Chevilly-Larue, France; ^3^ Legorreta Cancer Center at Brown University, Providence, RI; ^4^ Department of Biomedical Informatics, and Huntsman Cancer Center, University of Utah School of Medicine, Salt Lake City, UT; ^5^ Medical College of Wisconsin, Milwaukee, WI; ^6^ ProCan, Children's Medical Research Institute, The University of Sydney, Sydney, NSW, Australia; ^7^ The Kinghorn Cancer Centre, St Vincent's Hospital, Darlinghurst, NSW, Australia; ^8^ School of Clinical Medicine, Faculty of Medicine and Health, University of New South Wales, Sydney, NSW, Australia; ^9^ Jusidman Cancer Center, Sheba Medical Center, Ramat Gan, Israel; ^10^ Gray Faculty of Medical and Health Sciences, Tel Aviv University, Tel Aviv, Israel; ^11^ Department of Medicine, Brown University, Providence, RI; ^12^ Department of Biostatistics, Brown University, Providence, RI; ^13^ Avera Cancer Institute, Sioux Falls, SD; ^14^ Ben-Gurion University of the Negev, Be'er Sheva, Israel; ^15^ The Jackson Laboratory, The Maine Cancer Genomics Initiative, Bar Harbor, ME; ^16^ Vyas Cancer Research (VCR Park), Maharanipeta, Visakhapatnam, Andhra Pradesh, India; ^17^ Fudan University Shanghai Cancer Center, Shanghai, China; ^18^ Sir H.N. Reliance Foundation Hospital and Research Centre, Mumbai, India; ^19^ Burjeel Cancer Institute, Burjeel Medical City, Abu Dhabi, UAE; ^20^ Department of Medical Oncology, Dana-Farber Cancer Institute, Harvard Medical School, Boston, MA; ^21^ College of Medicine, Ras Al Khaimah Medical and Health Sciences University, Ras Al Khaimah, UAE; ^22^ Centre Hospitalier du Luxembourg, Luxembourg, Luxembourg; ^23^ University of Luxembourg, Luxembourg, Luxembourg; ^24^ Luxembourg Institute of Health, Luxembourg, Luxembourg; ^25^ King Hussein Cancer Center, Amman, Jordan; ^26^ National Center for Cancer Care and Research Hamad Medical Corporation, Doha, Qatar; ^27^ Department of Medical Oncology, Centre Léon Bérard, Lyon, France; ^28^ University of Lyon, Claude Bernard Lyon 1 University, INSERM 1052, CNRS 5286, Centre Léon Bérard, Cancer Research Center of Lyon, Lyon, France; ^29^ Department of Medical Oncology, INCLIVA Biomedical Research Institute, University of Valencia, Valencia, Spain; ^30^ CIBERONC, Instituto de Salud Carlos III, Madrid, Spain; ^31^ Catalan Institute of Oncology, Barcelona, Spain; ^32^ Federal State Autonomous Educational Institution of Higher Education I.M.Sechenov First Moscow State Medical University of the Ministry of Health of the Russian Federation, Sechenovskiy University, Moscow, Russian Federation; ^33^ Tata Memorial Centre, Affiliated to Homi Bhabha National Institute, National Cancer Grid, Mumbai, India; ^34^ University of Medicine and Pharmacy Iuliu Hatieganu, Cluj, Romania; ^35^ Academy of Medical Sciences, Bucharest, Romania; ^36^ Rutgers Cancer Institute, New Brunswick, NJ; ^37^ Vall d’Hebron Institute of Oncology (VHIO), Barcelona, Spain; ^38^ Vall d’Hebron Hospital Campus (VHIO), Barcelona, Spain; ^39^ Wake Forest University Health Sciences/Atrium Health (WFUHS), Winston-Salem, NC; ^40^ University of Vic—Central University of Catalonia, Vic, Spain; ^41^ Medical Oncology Department, Oncobell Program IDIBELL Institut Català d'Oncologia (ICO) Duran i Reynals Hospital, Clinical Sciences Department, Bellvitge Campus, University of Barcelona. CIBERONC, Barcelona, Spain; ^42^ CHAIM Medical Resource Organization, New York, NY; ^43^ School of Population and Global Health McGill University, Montreal, QC, Canada; ^44^ Beth Israel Deaconess Medical Center, Boston, MA; ^45^ Harvard Medical School, Boston, MA; ^46^ Homewood Research Institute, Guelph, ON, Canada; ^47^ University of Victoria, Victoria, BC, Canada

## Abstract

This review shares the ongoing work of the global Worldwide Innovative Network (WIN) Consortium for Precision Medicine to synthesize emerging cancer treatment data and to define the requirements for a common global cancer database that can truly support precision oncology. We performed a narrative review of emerging cancer treatment data, molecular profiling technologies, and existing clinicogenomic databases, focusing on how tumors are characterized, how subgroups are defined, and how demographic, lifestyle, and environmental factors are captured. The growth in molecular profiling technologies and the development of new targeted therapies are transforming cancer care. Tumors, regardless of tissue origin, are increasingly defined as composites of multiple, often rare, subgroups, each with distinct biology and likely response to specific therapies, based on multidimensional profiling of the tumor and its microenvironment. The solution lies in building vast databases that capture racial and ethnic diversity, reflected in genomic data, as well as diet and lifestyle factors that may have epigenetic impact on gene expression and post-translational modifications. A truly inclusive and informative data set must reflect global diversity, and there are multiple examples of demography-dependent differences in genomic signals. With members caring for and studying patients with cancer across five continents, WIN is actively exploring pathways to create a global cancer database, rich in clinical and molecular detail, granular enough for precise analysis, and large enough to power artificial intelligence–driven insights, provided appropriate data quality, validation, and governance frameworks are in place. This review surveys the current landscape and outlines practical paths forward to achieve this goal.

## INTRODUCTION

Cancer remains a leading global health challenge, with millions of new cases diagnosed annually.^1^ Despite advances, its molecular complexity, heterogeneity, and variable treatment responses demand continuous research and innovation. Traditional cytotoxic therapies adopt a one-size-fits-all approach that overlooks tumor molecular diversity, the microenvironment, and host immunity, yielding suboptimal outcomes and highlighting the need for personalized strategies.

Precision oncology (PO) offers a transformative approach by tailoring treatment to the molecular, immunologic, and microenvironmental characteristics of tumors. As cancers are increasingly recognized as collections of distinct and often rare subtypes, such as identifying patients' subsets whose tumors bear specific key prognostic and predictive biomarkers requires large, diverse, multi-institutional cohorts. However, limited access to such data sets remains a key barrier to clinical implementation and translational research.^2^ Although the use of targeted therapies, including immune-based treatments and antibody-drug conjugates, is expanding rapidly, their efficacy in early-phase trials is often restricted to narrowly defined molecular subgroups available in current databases, which results in limited ultimate access due to narrow regulatory approvals.

Overcoming these challenges requires a global infrastructure capable of integrating and analyzing large-scale, multi-dimensional data sets from diverse populations. Whether centralized or federated, it should harmonize demographic, molecular, and clinical outcome data, alongside key likely modifiers such as geographic origin, ethnicity, diet, smoking status, and environmental exposures,^3^ while ensuring confidentiality and data privacy remain paramount.

A major limitation of current cancer data sets is their lack of diversity, with underrepresentation of individuals of non-European ancestry. This restricts discovery of actionable mutations, and the implications of different genetic contexts, likely biases biomarker validation, and limits generalizability. As a result, therapies based on European-centric data may show different efficacy or unexpected toxicity in other populations. Developing globally representative databases could advance research and clinical translation by supporting artificial intelligence (AI)–driven trial design and real-world evidence (RWE) generation, and accelerating equitable, personalized treatments. Global efforts such as the World Economic Forum and the Global Alliance for Genomics and Health (GA4GH) highlight the urgency of inclusive, collaborative frameworks for advancing PO.^4^

## THE COMMON CANCER DATABASE

Cancer's genetic heterogeneity across populations underscores the need for global data integration to advance PO. Genetic mutations associated with cancer are not uniformly distributed but vary substantially by ethnicity and geography, influencing both disease biology and therapeutic response. For example, germline mutations in *BRCA1* and *BRCA2*, which increase the risk of breast and ovarian cancers and confer sensitivity to PARP inhibitors, demonstrate distinct distribution patterns across populations, directly affecting targeted therapy outcomes.^5^ In another example, non–small cell lung cancer (NSCLC) shows how population-level genetic variation can shape therapeutic development. In the Asia-Pacific region, *EGFR* mutations occur in up to 47% of NSCLC cases, substantially higher than in Western populations.^6,7^ This elevated mutation frequency has accelerated the development and adoption of EGFR-targeted therapies in these regions, an example of how regional genomic profiles can drive innovation in cancer treatment. Moreover, emerging evidence suggests that responses to targeted therapies, chemotherapy, and immunotherapy may also vary among ancestral populations.^8^

A major challenge in PO is the underrepresentation of diverse populations in genomic research, leading to disparities in diagnosis and treatment outcomes. A 2018 study reported that most genomic databases comprise data primarily from individuals of European ancestry, with African, Latin American, and Asian populations significantly underrepresented, limiting generalizability and equitable access to precision medicine.^9^ Similarly, a 2025 analysis found that approximately 95% of participants in genome-wide association studies were of European descent, reflecting a bias that reduces diagnostic and treatment accuracy and contributes to health inequities.^10^ Expanding diversity is also critical in the epigenetic space (transcriptomics, proteomics, lipidomics, and metabolomics) as well as in diet and lifestyle factors, which may influence antitumor efficacy and adverse event (AE) risks.^11^

### Benefits of a Global Database

A global cancer database would transform oncology by integrating diverse data sets to reveal patterns beyond those observed in smaller studies. This global scope is crucial for investigating rare cancers and uncommon or even rare molecular subtypes of common cancers, which require large cohorts for statistical validity. This platform would also accelerate discovery of tumor-agnostic therapies by identifying rare genomic alterations too uncommon for individual institutions to study. Through data harmonization and cross-cohort analysis, it would generate more generalizable findings, reduce research redundancy, and maximize the value of existing data sets. Ultimately, global data sharing would expedite therapeutic discovery and ensure more equitable, efficient use of research resources.

Integrating patient history into a global cancer database is essential for understanding care trajectories. Linking molecular, clinical, and real-world patient histories would improve assessment of therapeutic efficacy, AEs, and long-term outcomes, enhance predictive analytics, and accelerate the development of personalized treatment strategies. Such data could also support conditional and accelerated drug-approval models by enabling robust evaluation of targeted therapies in smaller molecularly defined cohorts.

Ultimately, patients will be the greatest beneficiaries of a unified global cancer database. Access to large, diverse, and well-curated data sets would enable clinicians to design therapies tailored to each patient's molecular and clinical profile, improving treatment efficacy and minimizing adverse effects. Such personalization would help close gaps in cancer care for underrepresented populations historically excluded from research.

### Challenges to Integration

Developing a unified global cancer database faces major challenges. A key obstacle is semantic and syntactic standardization, as data collected across institutions, regions, and languages vary in format, structure, and coding. Cancer centers also differ in data collection, process, and storage, using diverse sequencing platforms, electronic medical record systems, and documentation standards. Even institutions with comparable infrastructures capture data at varying levels of detail, and many use implicit or incomplete data dictionaries. Genomic and imaging data tend to be large, structured, and densely annotated, whereas clinical data are smaller, unstructured, and often sparsely annotated. Commercial systems are poorly suited to this heterogeneity, while federated solutions remain costly and nonstandardized.^12^ This variability and lack of explicit semantics complicate standardization, and harmonization may introduce inconsistencies or data loss, further increasing integration complexity.

Legal and ethical barriers pose major challenges to developing a global cancer database, as privacy and data-sharing regulations vary widely across jurisdictions. The European Union's General Data Protection Regulation imposes strict privacy requirements and limits cross-border transfers, restricting international collaboration. In the United States, the Health Insurance Portability and Accountability Act governs how patient data are stored, accessed, and shared, imposing strict obligations. Variation in anonymized data regulations, from unrestricted use to prohibition, creates inequalities, with some countries contributing data while others primarily benefit. Harmonized international standards could reduce these disparities and facilitate more equitable global collaboration.

Building and maintaining a global cancer database requires advanced infrastructure and strong governance. The system must securely manage large, heterogeneous data sets and ensure privacy during storage and transfer. Sustained investment, skilled personnel, rapid implementation of validated innovations, and transparent oversight are essential to maintain trust among patients and institutions. Differences in national laws raise data ownership issues, as institutions often control access even when patients consent to data use. Ethical concerns, particularly inconsistent consent procedures, further complicate data sharing. Harmonized consent frameworks and robust anonymization or pseudonymization are critical for secure, ethically compliant international collaboration.^13^

A major barrier to data integration is restricted access to clinical data held by pharmaceutical and biotechnology companies. These data are often unavailable during and even after trials conclude or study results are released, limiting independent validation and long-term outcome analysis. Concerns about intellectual property and competition contribute to limited data sharing, compounded by the absence of clear policies. These restrictions lead to redundant research and incomplete knowledge, as negative results remain underreported.^14^ Addressing this requires governance frameworks that balance commercial interests with ethical responsibilities, including the patients' rights to access their data, to maximize the utility of patient-contributed data. The pharma industry must consider whether the benefits from access to the global database, which will accelerate clinical trials and expand their generalizability to greater markets, outweigh their concerns for confidentiality.

### Addressing the Challenges

Overcoming these challenges will require collaboration among health care, technology, legal, and policy stakeholders. International partnerships are essential for creating shared standards and harmonizing ethical and legal practices across countries, while continued investment in infrastructure is critical to support a secure, efficient global database. Organizations such as the Worldwide Innovative Network (WIN) Consortium and the GA4GH demonstrate how coordinated efforts can advance cross-border data sharing.

Although collaboration provides the foundation, the main challenge lies in developing effective frameworks and protocols to ensure data compatibility, quality, and trust.^15^ Standardizing data collection across regions is a critical priority. International models such as the Observational Medical Outcomes Partnership (OMOP) Common Data Model (CDM), supported by the Observational Health Data Sciences and Informatics (OHDSI) initiative, offer a structured approach to organizing medical data and ensuring compatibility.^16^ By unifying clinical terminology and data structures, CDMs enhance the quality, comparability, and usability of integrated data sets.

AI technologies can facilitate data integration by automating the alignment of heterogeneous data sets, performing semantic harmonization, and enabling advanced analytics.^17^ Federated learning further allows institutions to collaborate without sharing raw patient-level data, preserving privacy and security.^18^

Methods such as retrospective harmonization and metadata cataloging can bridge gaps between diverse data sets. Together with flexible data-sharing agreements, sustainable funding, and strong partnerships among trial consortia, academia, health care organizations, and industry, these approaches can help build a unified global cancer database.

### Proposed Implementation Strategies

Developing flexible data-sharing agreements is essential for fostering international collaboration while protecting patient privacy. Adaptable legal frameworks enable cross-border data exchange, and involving legal experts ensures compliance with evolving regulations. Transparent policies build trust by clearly outlining the benefits and safeguards of data contribution. Balancing accessibility with strong privacy protections maintains confidentiality while supporting scientific progress. Standardized contract templates that define terms of use, responsibilities, protections, and proportionate consequences for material violations can help keep agreements effective as legal and technological contexts evolve. These agreements should also be supported by transparent monitoring and audit mechanisms to promote accountability across participating sites.

Securing funding from governments, philanthropic organizations, and the private sector is essential to develop and sustain a global cancer database with strong data management, security, and compliance systems. Coordinated international funding can support participation from developing countries and improve equitable global access to research and treatment. Once pharmaceutical companies appreciate the value to them of such an enormous and comprehensive noncompetitive space as this database, they may well contribute to building this global database, which will them with landscaping information to inform drug development and marketing strategies.

### Minimum Data Set and Data Model Strategy

To support practical implementation while preserving flexibility, we propose a minimum viable data set as a nonprescriptive foundation for the WIN common global database. This data set focuses on a core set of harmonized domains necessary for clinical relevance and cross-site interoperability. These include, for example, (1) patient demographics (age, sex, ethnicity, and geographic region); (2) tumor characteristics (cancer type, histology, stage, date of diagnosis, and key molecular subtype); (3) treatment exposures (systemic therapies, procedures, and lines of therapy with start and end dates); (4) outcomes (vital status, survival end points, response assessments, and AEs where available); (5) key biomarkers (actionable genomic alterations and testing modality); and (6) longitudinal follow-up time points capturing disease trajectory. The iterative refinement of this minimum data set and broader implementation framework may be supported by multidisciplinary working groups developing practical outputs such as data dictionaries, governance recommendations, template consent elements, and implementation guidance for diverse regulatory and resource settings.

We envision the OMOP CDM as the backbone for standardized clinical data harmonization and federated analytics. However, OMOP alone cannot accommodate the scale and complexity of multiomics and imaging data. These data types would be managed through linked external repositories or object stores, with structured metadata indexed within OMOP to enable discoverability and integration. Interoperability across institutions can be facilitated through standards such as Fast Healthcare Interoperability Resources, supporting data ingestion and exchange from heterogeneous clinical systems.

Importantly, this framework is intended as a starting point for iterative refinement through a stakeholder-driven consensus process, allowing adaptation to local constraints while maintaining global interoperability. At a second step, the database will add both health economics information and patient experience data, using rapidly evolving instruments,^19^ to begin to integrate value-based care into the system of cancer research and treatment.^20,21^

### Governance Framework and Stakeholder-Driven Implementation Pathway

Building a global cancer database requires governance that is clear, fair, and trusted across different legal and health system contexts. Rather than defining a fully detailed governance model initially, WIN will build on its long experience of a global stakeholder-driven, consensus-based process to develop and refine its framework.

WIN already has experience and success at convening key stakeholders, including academic institutions, health care providers, patient groups, industry partners, and representatives from low- and middle-income countries (LMICs) for collaborative efforts in clinical research efforts, and can use this to guide implementation of core governance elements, aligned with international standards and regional requirements. WIN's data science subcommittee has initiated these considerations.

Core governance components include distributed data stewardship, so data remain under the control of contributing institutions; transparent access governance through a data access committee that reviews requests for scientific merit, ethics, and equitable representation; fair attribution and publication practices; and equitable benefit-sharing, particularly for LMIC partners. Equitable benefit-sharing includes priority access to aggregated analytics, coauthorship guidelines, and targeted capacity-building grants for LMIC partners. We also explicitly align this framework with established international standards, including GA4GH policy tools. Although not intended as a finalized governance model, this addition provides a concrete and actionable foundation aligned with the collaborative and evolving nature of this effort.

To translate this vision into practice, we propose a stepwise, federated implementation pathway that balances feasibility with global inclusivity. Initial efforts will focus on developing a shared metadata catalog and harmonized data dictionary across participating institutions, followed by pilot federated initiatives across 3-5 sites and selected tumor types, with success defined by query execution volume and cross-site interoperability metrics, leveraging WIN disease committees as operational entry points. Subsequent phases would expand interoperability, analytic capabilities, and integration into clinical workflows such as molecular tumor boards (MTBs) and RWE generation.

The feasibility of such an approach is supported by prior experience in pediatric oncology, where global platforms such as Cure4Kids and POND4Kids have demonstrated that federated, incrementally developed data-sharing ecosystems can achieve broad international adoption, including in LMIC settings.^22,23^ These initiatives highlight the value of contextual adaptability, sustained investment in local data infrastructure, and governance models grounded in trust and shared clinical purpose. Furthermore, illustrative national and LMIC case examples underscore the feasibility and contextual adaptability of this vision (Appendix 1 and Appendix 2 in the Data Supplement).

### Technological Consideration for Data Integration

Technological advances offer powerful tools to address the logistical and technical challenges of building a global cancer database. Cloud platforms provide scalable, cost-effective infrastructure for real-time data access and collaboration. In regions with strict privacy regulations, decentralized or federated models can mitigate concerns by allowing institutions to analyze data locally while sharing only aggregated results. This model, however, requires each institution to maintain adequate technical capacity, which may necessitate rebalancing resources through targeted investment and capacity-building support from governments, international organizations, and philanthropic foundations, particularly for partners in low-resource settings.

AI and machine learning further enhance data analysis, identifying patterns and correlations often missed by traditional methods, although their performance depends heavily on data quality, representativeness, and appropriate validation frameworks. These tools accelerate treatment discovery and refine therapies using RWE. However, their effectiveness depends on access to sensitive patient-level data, raising significant privacy concerns. Even anonymized data sets may risk reidentification when linked with other sources. Approaches such as federated learning^24,25^ and differential privacy^26,27^ can help safeguard information while preserving data utility.

Realizing the potential of AI in this context requires three safeguards. First, data quality gates must be established to ensure reliability of downstream analyses, including standardized curation procedures, completeness thresholds for key variables, harmonized definitions of clinical end points, and mechanisms to detect and mitigate label noise from inconsistent annotation. Given the heterogeneity of contributing institutions, continuous quality monitoring and provenance tracking are essential. Second, bias auditing and fairness assessment are necessary to ensure that AI models trained on global data sets do not perpetuate or amplify existing disparities. Differences in data capture across regions, health care systems, and populations may introduce systematic biases; evaluating model performance across demographic, geographic, and clinical subgroups is a critical step toward equitable AI. Third, robust external validation is required before clinical deployment, including evaluation across independent data sets from multiple institutions and geographic regions, ongoing monitoring for model drift, and transparent reporting of performance metrics. Embedding these principles into the design of a global cancer database will help ensure that AI-driven insights are reliable, reproducible, and clinically actionable.

Table 1 summarizes the committee's identified challenges and proposed solutions.^28-32,35,36^

**TABLE 1:** Summary of Challenges and Their Potential Solutions

Category	Challenge	Potential Solutions	Ongoing Efforts
Ethical, legal, and privacy	Privacy EU regulations (GDPR), US regulation (HIPAA), and other countries' rules compatibility Preventing identifiers' leakage; using data for multiple studies	Adopt the more stringent regulation: GDPR. Requires an EU affiliate for non-EU organizations. GDPR regulations; routine request for general data use consent from all patients; decentralized databases	Efforts at WIN and individual centers to address policy or local laws to facilitate research. For example, in the State of RI, US, in 2024, a data sharing law was passed to allow sharing of deidentified data. In 2024, the WIN Consortium created a policy committee and is working with patient advocacy organizations to maximize patient voices and interests
Technical issues	Collect multiple types of data, transfer, and storage of large amounts of data	Data standardization and harmonization, scalable data storage, and data management platforms	International collaborations including WIN Consortium and others working to address harmonization
Data entry variabilities	PROMs, EMR structured fields, EMR free text, laboratory results, imaging, pathology, gene sequencing, and socioeconomic data	Linking to distinct databases at each site, for both PROMs and economic data, and data mappings	PMT database in Canada linking to provincial costing databases and PROM databases. Collaborations to further refine essential data elements such as USCDI+ Cancer
Dictionary harmonization	Lack of standardized clinical terms	Use codebooks and data dictionaries	FHIR; openEHR; OMOP; OHDSI
Lack of broad population representation	More databases in Western affluent medical centers	International efforts of inclusion, intentional inclusion of unrepresented populations, proactive outreach, and community engagement initiatives	ATLAS,^28^ All of Us,^29^ Genomics England,^30,31^ H3Africa^32^
Data ownership	Patient-owned data; medical center–owned data; country-owned data; clinical trial data	Decentralized databases; federated learning	Collaborations strive to secure permissions for data use to facilitate progress
Genomic data	High complexity; several levels of data (sequence/gene/targeted mutations); privacy concern heightened	Federated learning, AI-based secure multiparty computation, privacy-preserving methods (eg, differential privacy)	TCGA, Genomics England, and others
Radiologic data and pathologic data (whole-slide images)	Exceptionally large data files; AI-based evaluation technology not yet mature	Use structure radiology reports; develop organ/pathology-focused LLM	Opportunities with cooperative groups such as ECOG-ACRIN^33^
Clinical data	Mostly recorded as free text	Encourage use of structured fields; LLM	^34^
Treatment efficacy documentation	RECIST not used in clinical practice; lead-time bias issues; crossover and subsequent treatments affect OS in a less-controlled manner; patient and tumor target heterogeneity; end points are challenging to document within real-world data	Surrogate measures of efficacy (TNT, survival); LLM evaluation of response from imaging	Validation of surrogate measures; PFS2/PFS1 N-of-1 trial designs
Longitudinal data	Lack of unique patient identifiers across health institutions	Unique health-related identifiers exist in some countries (eg, Israel); federated linking methods, probabilistic matching techniques, and blockchain-based identifier solutions	Similar to data entry variables; national identifier systems in European countries (Denmark CPR,^35^ Sweden PIN^36^)

Abbreviations: AI, artificial intelligence; ACRIN, American College of Radiology Imaging Network; CPR, Central Personal Register; ECOG, Eastern Cooperative Oncology Group; EHR, electronic health record; EMR, electronic medical record; EU, European Union; FHIR, Fast Healthcare Interoperability Resources; GDPR, General Data Protection Regulation; HIPAA, Health Insurance Portability and Accountability Act; LLM, large language model; OHDSI, Observational Health Data Sciences and Informatics; OMOP, Observational Medical Outcomes Partnership; OS, overall survival; PIN, Personal Identity Number; PFS1, progression-free survival (for treatment 1); PFS2, progression-free survival (for treatment 2); PMT, Personalize My Treatment; PROMs, patient-reported outcome measures; TCGA, The Cancer Genome Atlas; TNT, time to next treatment; US, United States; USCDI, United States Core Data for Interoperability; WIN, Worldwide Innovative Network.

### Similar Efforts

Numerous large-scale cancer genomics initiatives have created multicenter databases, providing important lessons in data collection, harmonization, and sharing across diverse health care systems. However, none yet capture a truly global population, and representation from LMICs remains limited. Tables 2 and 3 summarizes, respectively, several major efforts^37-43^ and key areas where collaboration and integrated data systems could drive progress.^11,44-60^

**TABLE 2:** Summary of Major Multi-Institutional Cancer Genomics Initiatives

Project	Description
TCGA^37,38^	One of the most widely used data sets for cancer research, providing comprehensive multiomic genomic profiles for over 11,000 tumors spanning 33 cancer types. Although TCGA covers a broad range of tumors, the cohort primarily comprises US-based patient samples, with earlier-stage disease disproportionately represented
GENIE^39^	An AACR international pan-cancer registry that aggregates real-world clinical-grade genomic data from participating cancer centers. Unlike TCGA, which primarily includes research-grade data from treatment-naïve tumors, GENIE captures data from patients treated in routine clinical settings, including advanced and metastatic disease. This real-world data approach enables longitudinal tracking of treatment outcomes and resistance patterns across diverse populations and cancer types. Although GENIE aspires to be international, its current data set is heavily skewed toward US-based patients, which can limit global generalizability somewhat—similar to TCGA
Genomics England—100,000 Genomes Project^30,31^	A UK initiative primarily focused on rare diseases and cancer. It integrates whole-genome sequencing data with detailed clinical records for participants recruited through the NHS. This project places considerable emphasis on nationwide coverage across England
ATLAS^28^	A notable initiative aiming to unify oncology research efforts across multiple Asian countries. By coordinating clinical trials, sharing genomic profiles, and aligning treatment protocols, it strives to address regional disparities in care and outcomes. With a network of medical centers in countries such as Japan, South Korea, Singapore, and Thailand, this project enhances cross-border collaboration and aims to improve the representation of Asian populations in large-scale cancer studies
ICGC-ARGO^40,41^	A next-generation international initiative building upon the original ICGC. It represents a global effort involving multinational partnerships designed to integrate extensive genomic data with detailed clinical annotations, aiming to enhance precision oncology. ICGC-ARGO combines newly generated prospective genomic data with existing data sets from ICGC to facilitate large-scale cross-cohort analyses. Supported by numerous countries, ICGC-ARGO aims to characterize tens of thousands of patient genomes, enhancing diversity and global representation to address cancer heterogeneity and ultimately advance precision medicine worldwide. Although ICGC-ARGO aims for global representation, most contributing institutions are from high-income countries. Populations from lower- and middle-income countries and underrepresented ethnicities remain comparatively less represented, potentially limiting the generalizability of findings
1+MG Initiative^42^	This initiative, launched in 2022 and still under development, aims to create a federated, secure infrastructure for genomic and clinical data access across Europe. The initiative spans European Union countries and faces challenges such as language diversity and heterogeneity in data annotation. Although English is commonly used, inconsistencies often referred to nonstandard or unclear usage can hamper clear communication. Another major hurdle is the lack of harmonized standards, particularly for clinical data, which is more complex than genomic data to align. The diversity of health care systems across the European Union further complicates integration, with examples such as Spain's different regional health care systems each using its own medical record formats
All of Us^29^	A NIH initiative aiming to accelerate health research by enabling individualized prevention, treatment, and care, with a strong focus on including populations historically underrepresented in medical research. The program collects data exclusively from participants within the United States and is not limited to cancer; it covers multiple conditions, including healthy individuals. As of early 2025, over 848K participants have enrolled. The program curates a wide variety of data types, including survey responses, EHRs, physical measurements, genotyping arrays, whole-genome sequencing (both short- and long-read), and wearable device data. *All of Us* standardizes its EHR data to the OMOP CDM, allowing integration across diverse data sets. Although it provides one of the largest and most comprehensive collections of genomic and phenotypic data, limitations exist—for instance, no gene expression or protein-level data are currently available, and EHR data may not be complete for all participants
SPHN^43^	A national Swiss initiative to enable responsible, privacy-conscious health data use for research. SPHN promotes the development of an interoperable data ecosystem across Swiss hospitals and research institutions. It operates under a federated model where data remain at the source but can be accessed through standardized frameworks, thus supporting both clinical and genomic research efforts within Switzerland
CINECA^32^	An initiative that focuses on building a federated data-sharing infrastructure to enable interoperability, especially in cancer and rare disease research. It connects with other initiatives such as 1+MG, SPHN, and H3Africa, enhancing global collaboration efforts
EUCANCan	A project that focuses on harmonizing cancer genomics workflows across different institutions. It facilitates data access across borders by using federated models, bringing together hospitals, academic centers, and biobanks from Europe and Canada to support standardized cancer genomics research
Caris POA	As of 2025, the database contains RNA-seq data from ∼500,000 patients with cancer across cancer types. The database contains genomics, demographics, molecular targets, drug treatments, and patient outcomes. A number of WIN Consortium members are members of the Caris POA. The Caris POA database generates Kaplan-Meier curves to compare defined cohorts, volcano plots to associate trends among altered genes, and can extract subset data (eg, immune signatures) from bulk RNA-seq data
Cure 51	WIN member Cure 51 has been creating a database in different tumor types such as lung cancer, glioblastoma, pancreatic cancer, and others to understand exceptional responders and molecular characteristics that associate with the clinical outcomes

Abbreviations: 1+MG, 1+ Million Genomes; AACR, American Association for Cancer Research; ARGO, Accelerating Research in Genomic Oncology; ATLAS, Asian Clinical Trials Network for Cancers Project; Caris POA, Caris Life Sciences Precision Oncology Alliance; CDM, Common Data Model; CINECA, Common Infrastructure for National Cohorts in Europe, Canada, and Africa; EHR, electronic health record; EUCANCan, European Canadian Cancer Network; GENIE, Genomics Evidence Neoplasia Information Exchange; ICGC, International Cancer Genome Consortium; NHS, National Health Service; NIH, National Institutes of Health; OMOP, Observational Medical Outcomes Partnership; SPHN, Swiss Personalized Health Network; TCGA, The Cancer Genome Atlas; UK, United Kingdom; WIN, Worldwide Innovative Network.

**TABLE 3:** Topics Requiring Multi-Institutional Integrated Databases and Collaboration That Represent Opportunities in Advancing the Field of Precision Oncology

Goal	Ongoing Examples	Examples of Solutions
Quality assurance	Multi-institutional cancer databases are using rigorous data quality checks to ensure reliable insights. For example, the AACR Project GENIE Biopharma Collaborative established a scalable QA process for curating oncology electronic health records across sites.^44^ These efforts emphasize standardized data collection and validation to minimize errors and variability^44^	Broad adoption of automated data curation pipelines and unified data standards can further improve quality. Developing shared, open-source workflows for data preprocessing and quality control (as demonstrated in a recent multiomics platform) helps ensure high-quality, comparable data sets across institutions^45^
Creating real-world control groups	Real-world data are increasingly used as synthetic control arms in clinical trials and regulatory submissions. For instance, about 17% of recent EMA oncology drug approvals (2016-2021) incorporated external control cohorts drawn from real-world data, illustrating growing acceptance of this approach.^46^ Such examples show that real-world cohorts can contextualize single-arm trials, although methodological challenges remain^46^	Advancing this area will require careful study design and analytic rigor to build credible external controls. Experts recommend prospectively planning for external controls, ensuring baseline patient comparability, using consistent outcome measures, and applying robust statistical methods to mitigate bias.^46^ Collaborative efforts between sponsors and regulators to develop clear frameworks and guidance will also strengthen the use of real-world control arms
Identifying efficacy predictors	Precision oncology studies are actively identifying biomarkers that predict which patients will benefit from therapies. For example, a recent analysis found that tumors with elevated mTORC1-driven protein translation had better responses to the AKT inhibitor capivasertib in a cohort of *PIK3CA*-mutated cancers, demonstrating how molecular features can correlate with drug efficacy.^11^ Ongoing trials often include correlative studies to link genomic or proteomic markers with treatment outcomes, yielding predictive signatures^11^	To further improve efficacy prediction, researchers are calling for biomarker-driven trial designs and integrative data analysis. Strategies include enriching clinical trials with patients most likely to respond based on molecular profiling and using multiomic and computational models to discover robust predictive signatures.^47^ Greater sharing of data across studies and the use of AI/machine learning can help identify complex combinations of biomarkers that reliably forecast treatment success^47^
Identifying toxicity predictors and resistance	Researchers are also seeking biomarkers to predict treatment toxicity before therapy begins. For instance, a germline microRNA-based signature was shown to predict which patients would develop grade ≥2 immune-related adverse events on anti–PD-1/PD-L1 checkpoint inhibitors, regardless of cancer type.^48^ Such findings illustrate the potential of genetic and molecular factors to serve as early warnings for severe toxicities.^48^ Additionally, extensive research is underway to characterize genomic and transcriptomic markers associated with therapeutic resistance, such as secondary mutations in *EGFR* or immune checkpoint blockade resistance mechanisms involving tumor microenvironment modulation^49–53^	Progress in this area can be achieved by integrating patient-specific genomic and clinical data into toxicity risk models. Experts suggest embedding pharmacogenomic analyses in trials and real-world studies to uncover variants or profiles associated with adverse outcomes and resistance mechanisms.^47,54^ Improving data sharing on treatment side effects and applying machine learning to large patient data sets could enable the development of decision support tools that flag high-risk patients before toxicity occurs.^47^ Improving data sharing on treatment side effects and resistance outcomes, coupled with applying machine learning techniques to large patient data sets, could enable the development of robust decision support tools.^55–57^ These tools would help clinicians preemptively identify patients at high risk for toxicity or resistance, guiding personalized treatment adjustments
Landscaping studies	Large-scale landscape initiatives are mapping cancer genomics and outcomes across populations. The Cancer Genome Atlas was an early example, and more recently, the AACR Project GENIE consortium has aggregated over 100,000 tumor cases from multiple hospitals to enable pan-cancer analyses of genomic alterations and their frequencies.^39^ These collaborative data sets provide comprehensive views of mutation and biomarker landscapes across diverse cancers, helping researchers identify patterns that would be indiscernible in smaller, single-institution studies^39^	To advance landscaping studies, expanding global data representation and data types is crucial. Efforts such as the All of Us Research Program are underway to increase the diversity of genomic data by enrolling participants from underrepresented populations, which will improve the generalizability of landscape analyses.^29^ Additionally, international projects are defining standard minimal data sets for oncology (eg, the 1+ Million Genomes initiative) to harmonize data collection, and continued integration of multiomic data (genomic, transcriptomic, and proteomic) will yield richer, more actionable cancer atlases
Creating AI decision support tools	AI-driven clinical decision support tools are being developed to assist oncologists in treatment planning. For example, the xDECIDE platform (from the company xCures) demonstrates an AI-augmented system in which a human-AI team analyzes a patient's medical records and recommends personalized cancer treatment options, with outcomes tracked to continually improve the model.^58^ Similarly, prototype oncology AI systems (including large language model–based assistants) have shown promise in synthesizing research knowledge to suggest therapies, although they are not yet widely deployed in practice^58^	Realizing the full potential of AI decision support will require validation, integration, and trust-building. Researchers emphasize the need for rigorous clinical validation of AI recommendations against standard care outcomes, as well as user-friendly integration of AI tools into clinical workflows.^2^ By investing in high-quality training data and addressing biases (so that AI recommendations are equitable and evidence-based), the goal is to deploy AI copilots that can reliably help clinicians navigate complex data and treatment choices by 2030^2^
Identify various SOCs across the world	Global analyses reveal wide variation in cancer standards of care and outcomes across different regions. For example, one recent study highlighted striking disparities in the availability and cost of cancer medications: Many newer therapies are far less accessible in low-income countries, contributing to worse outcomes compared with high-income countries.^59^ Differences in health care infrastructure—from access to screening and diagnostics to the timeliness of adopting new treatments—mean that the standard care for a given cancer can vary greatly between countries or even within regions	Closing these gaps will require international collaboration and policy initiatives to elevate standards of care everywhere. Solutions proposed include increasing the use of quality-assured generic drugs and biosimilars to make treatments^60^ affordable, implementing universal health coverage to reduce financial barriers, and investing in health care infrastructure and training in low-resource settings.^59^ Targeted programs can also improve specific capacities—for instance, establishing frameworks to expand advanced diagnostic imaging in Africa^60^—thereby bringing global standards of cancer care more in line with best practices
Study rare patient populations/identify novel tumor drivers/vulnerabilities	Integrative data efforts help study rare cancers and discover new tumor vulnerabilities that single institutions cannot easily capture. The ICGC-ARGO international project, for example, focuses on rare and familial cancers (such as certain hereditary pancreatic cancers) by pooling genomic and clinical data worldwide, which has shed light on unique tumor drivers and informed precision medicine approaches for these uncommon cases.^40^ Similarly, large consortia and databases enable identification of rare oncogenic mutations or exceptional responders	Develop targeted multi-institutional registers and databases to systematically collect and analyze rare cancer cases. Leverage international genomic data-sharing consortia (eg, ICGC-ARGO, GENIE) to aggregate diverse patient data sets, facilitating identification of novel molecular drivers and potential therapeutic targets for rare cancers and exceptional responders

Abbreviations: AACR, American Association for Cancer Research; AI, artificial intelligence; ARGO, Accelerating Research in Genomic Oncology; EMA, European Medicines Agency; GENIE, Genomics Evidence Neoplasia Information Exchange; ICGC, International Cancer Genome Consortium; QA, quality assurance; SOC, standards of care.

Despite these advances, many LMICs still face major barriers to contributing robust cancer and medical imaging data, including limited infrastructure, resources, and technical expertise. The Consortium for Advancement of Magnetic Resonance Imaging Education and Research in Africa (CAMERA)^60^ illustrates how targeted programs can help. Through the SPARK (Sprint Artificial Intelligence Training for African Medical Imaging Knowledge Translation) Academy, CAMERA supports AI training and case-based learning to build local capacity and reduce resource constraints.

## CURRENT WIN DATA SETS AND VISION

**FIG 1:**
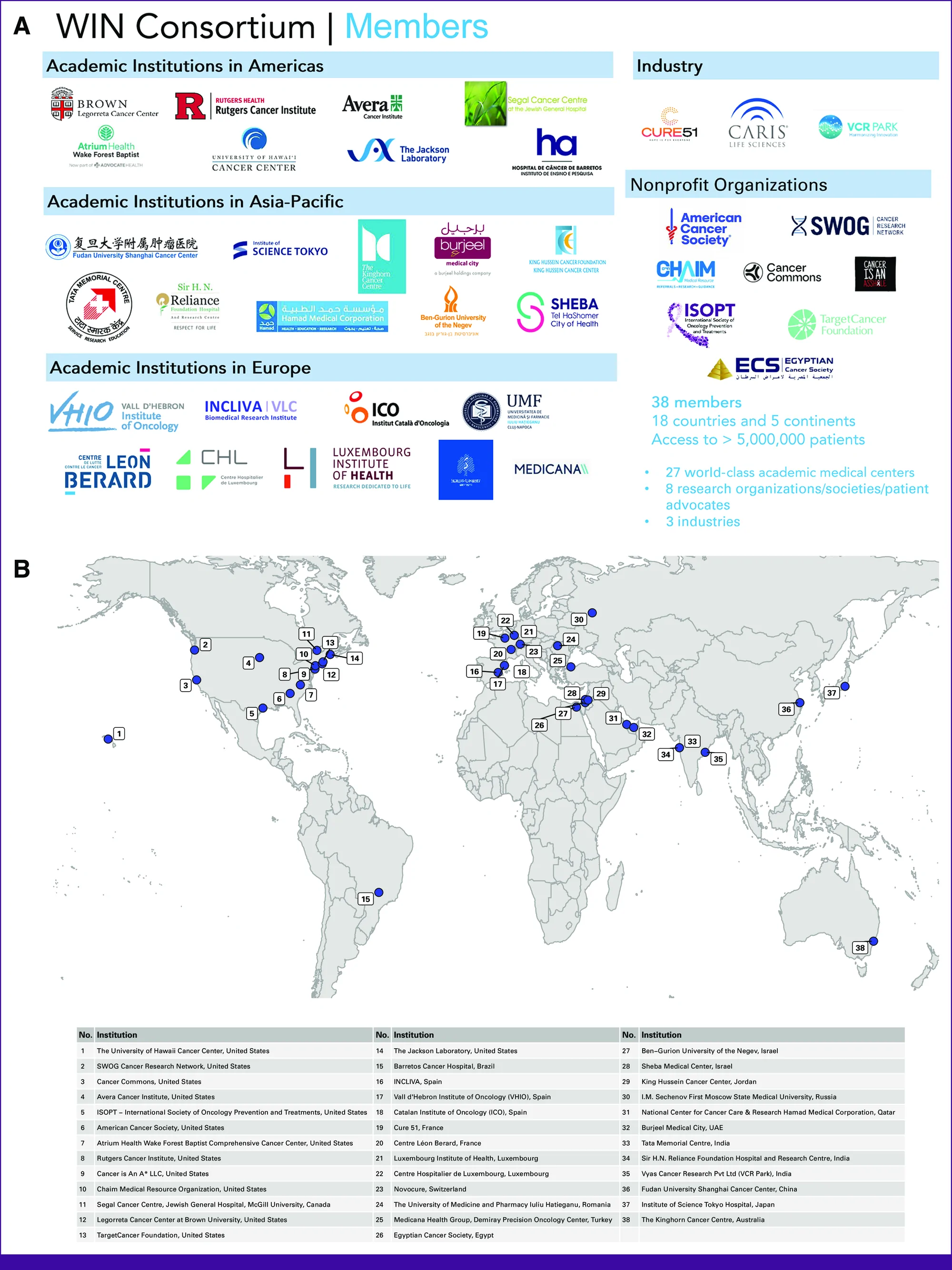
The WIN consortium members. (A) WIN consortium's world-class academic medical centers, SME, research, and patient advocacy organizations. (B) Geographic distribution of WIN consortium members around the world. SME, small and medium enterprise; WIN, Worldwide Innovative Network.

In the effort to create global, large-scale data sets, the WIN Consortium unites institutions worldwide as outlined in Figure 1, each maintaining repositories of genomic, clinical, and pharmacogenomic data. Data science committee discussions highlighted major heterogeneity across testing platforms, data structures, depth of annotations, longitudinal follow-up, and privacy requirements, all of which complicate cross-border interoperability. Table 4 summarizes data sets currently available across WIN member institutions.^61-63^

**TABLE 4:** Data Availability at WIN Consortium Participants

Institution/Participant	Data
Atrium Health Levine Cancer (United States)	Performs molecular profiling for most patients with third-party vendors (Tempus, Caris, etc), complemented by in-house panels; data available in multiple formats: CSV, JSON, BAM, FASTQ; database (SQL) with clinical genomic profiling + trial matching from in-house LInK program; multiple disease site–specific databases with clinical and molecular data plus germline database (clinical tests); specimen database integrated with registry data; over 40,000 new patients a year across five states at this time; integrated clinical-genomics database for system in progress; piloting AI system for augmenting research database; part of AACR GENIE
Avera Cancer Institute (United States)	Uses commercial partners and can access discrete and raw data. Performs in-house testing for small panels and pharmacogenomics. Maintains a curated MTB database covering 10+ years, 3.5K patients and comprehensive data on treatment, sequencing, AEs, and outcomes. Multi-omics platform study is actively recruiting 1K+ patients enrolled with tissue sequencing, serial liquid biopsies, pharmacogenomics, microbiome, SDOHs, and deep EHR annotation (via REDCap). EMR mining is ongoing but challenging. Aims to migrate to OMOP
Burjeel Holdings (UAE)	Maintains all clinical data (diagnostics, treatments, and outcomes) on EMR (>5,000 patients/year) The group provides comprehensive central laboratory services for all hospitals, medical centers, and clinics of BH network. BH also has an integrated clinical and laboratory information system (Cerner Millennium) with high level of connectivity. BH's expansive clinical expertise with multiple institutions such as Burjeel Cancer Institute (with medical, surgical, radiation, and palliative oncology services), bone marrow and solid transplant programs, state-of-the-art imaging facility, and OncoHelix-Colab (in-house molecular diagnostics laboratory) creates a unique opportunity for future-oriented precision oncology and AI-based programs
Catalan Institute of Oncology (Spain)	Holds genomic data (15K germline NGS samples, 1.5K somatic 500-gene panel) from Illumina-based assays on in-house developed database Stores both germline and somatic complete data from NGS panels using unstructured files on a data storage server. Migration to a data lake is planned. In the germline context, an ad hoc panel of approximately 150 DNA genes is used, including main hereditary cancer genes and others with low penetrance for research. For somatic determinations, a commercial panel (TruSight Oncology 500 from Illumina) is used, which includes 523 DNA genes and multiple RNA variant types (single-nucleotide variants, copy-number variants, indels, and fusions), plus microsatellite instability and tumor mutational burden. Results are mainly structured in an in-house relational database developed for storing and querying variants' results. Semiautomatized extract, transform, load processes are usually used to query the complete or specific molecular results. Final informed results are presented in PDF reports and clinical data mainly stored as electronic medical records in the hospital information system Clinical data are stored in the hospital registry that fulfills all registry regulatory requirements
Centre Hospitalier de Luxembourg/Luxembourg Institute of Health (Luxembourg)	Uses commercial and Luxembourg public laboratory partners (Onco DNA and LNS) and can access raw data from some. Maintains curated data (treatments, sequencing, AEs, and outcomes) on patients in MTB program over 10 years (200 patients). Mining EHRs is ongoing but challenging
Centre Léon Bérard (France)	ConSoRe is a powerful federated data mining platform deployed across the UNICANCER network of comprehensive cancer centers. Using natural language processing, ConSoRe structures and harmonizes data within a common data model, enabling the creation of patient cohorts based on tumor characteristics, patient profiles, and treatment modalities. At Centre Léon Bérard, ConSoRe aggregates data from over 360K patients with cancer and more than 18M medical records that can be integrated with results from in-house genomic panels, whole-exome, whole-genome sequencing, and RNA sequencing from patients enrolled in various molecular profiling initiatives, including the PROFILER studies and France Médecine Génomique 2025. Findings are reviewed in a high-volume molecular tumor board, where AI-driven multiagent systems are also evaluated
Fudan University Shanghai Cancer Center (China)	Has independently developed over 30 multiple-genes examination products, covering major cancer and simultaneously detecting somatic and germline mutations, insertions and deletions, CNVs, gene fusions, MSI, and TMB in tumors. Both germline and somatic complete data from NGS panels are stored on a data storage server. And results are mainly structured in an in-house relational database developed to store and query variants results. Final informed results are presented in PDF reports and clinical data are mainly stored as electronic medical records at hospital information system Clinical data are stored in the hospital regulatory-compliant registry The cumulative number of patients tested has exceeded 75,000 cases from 2018 to 2024
Hamad Medical Corporation (Qatar)	The Qatar Precision Oncology Collaborative has been established at the NCCCR as a partnership between NCCCR/Hamad Medical Corporation and researchers at QBRI. The initiative focuses on breast and thyroid cancers diagnosed in Qatar from 2015 to 2022, maintaining curated clinical, treatment, and outcome data. Efforts are underway to collect tissue samples from these patients for comprehensive molecular profiling—including RNA/DNA sequencing, genomic panels (TMB/MSI and somatic mutations), prediction analysis of microarray 50 risk of recurrence scoring, and spatial immunoprofiling—to correlate molecular findings with patient survival outcomes
I.M Sechenov First Medical State University (Russia)	Has a database of 1,000 tumor exomes with clinical data annotation
INCLIVA Biomedical Research Institute (Spain)	Primarily holds genomic raw data from solid tumors (Oncomine). In-house–developed database for storing data from the Thermo Fisher–based assays. In 2024, 838 patients had next-generation sequencing. However, the in-house–developed database included basic clinical data such as age, sex, tumor location, diagnosis, and biopsy type
Institute of Science Tokyo Hospital (Japan)	Profiles solid tumor patients using commercially available CGPs (FoundationOne CDx, FoundationOne Liquid Cdx, Guardant360 Cdx, and local panels). Majority of patients are Japanese. Clinical and genomic data are available for analysis. Also, has access to approximately 100,000 clinicogenomic data for 100,000 patients in Japan
Jewish General Hospital/Exactis (Canada)	Developed PMT for clinical trial matching, covering 11K patients at 16 hospitals in 6/10 provinces to date. PMT includes longitudinal data on solid tumors
King Hussein Cancer Center (Jordan)	A hospital-based cancer registry was established in 2006 and holds data on over 70K patients with cancer. Additionally, there are germline mutation data on over 10K patients with solid tumors
Kinghorn Cancer Centre, St Vincent's Hospital/Garvan Institute of Medical Research (Australia)	Holds clinical and demographic and response data to 3,000+ patients referred for early-phase clinical trials since 2017. One third of patients had genomic profiling performed via commercial and academic panels but are not integrated into this database and raw data are not available. Also holds MGRB, comprising whole-genome sequence and phenotype of ∼4,000 elderly Australians depleted for cancer, cardiovascular disease, and dementia
Legorreta Cancer Center at Brown University (United States)	Numerous projects that involve the Caris POA database. Molecular modeling (proteins and cancer-related pathways) and AI-based approaches. Requires trajectory/confirmational data and potential integration with genomic data. Major initiative in RNA modifications and biology in general and specifically in cancer
The Jackson Laboratory, Maine Cancer Genomics Initiative (United States)	The MCGI Registry serves as a data resource for the scientific community for future cancer-related genomic medicine research. The Registry contains information related to a participant's cancer diagnosis (curated pathology and clinical data), cancer treatment data, tumor sequencing results (curated NGS data from commercial and in-house providers), and survival (up to 12 months), as well as demographic and patient-related outcomes data. Registry accrual started in 2017 and is currently at ∼1,800 patients. The data are made available governed by a data access committee for IRB-reviewed research projects
ProCan, Children's Medical Research Institute (Australia)	Partners with over 100 groups globally to add proteomic data to retrospective cohorts (clinical trials or real-world). Over 30K samples with deep proteomic profiling and well-annotated clinical and outcome data along with matched normal tissue samples included (for selected cases). Maintains an in-house unified database with clinical and proteomics data for cross-cohort queries
Rutgers Cancer Institute (United States)	Part of ORIEN. Uses commercial partners (Foundation Medicine, Caris, Guardant, etc) and can access raw data. Conducts in-house testing with smaller panels. In-house and commercial data linked to biorepository specimens and clinical data through an in-house clinical data warehouse (∼200,000 patients and ∼10% of these have genomic tests). Weekly molecular tumor board for 10+ years with treatment recommendations and referrals for clinical trials
Sheba Medical Center (Israel)	OncoMindRWD project automatically extracts/transforms/loads clinical, pathologic, radiologic, laboratory, and treatment data to a single SQL database. Large language models are being integrated for efficient and accurate data extraction from free text as well as for ETL of laboratory data of a variety of sources
Sir H. N. Reliance Foundation Hospital, Mumbai (India)	The precision oncology center holds data from 5,000 patients—including treatments administered, diagnostic tests performed, sequencing results, and clinical responses. The data are systematically recorded in the internally managed EMR system that is readily accessible to all treating physicians for seamless clinical decision making. Mostly collaborates with commercial partners such as Datar Cancer Genetics, Strand Life Sciences, Karkinos Healthcare, Foundation Medicine, Tempus, Guardant Health etc for advanced molecular diagnostics. Other small, in-house–developed panels or panels developed in collaboration with molecular partners may also be used occasionally
Tata Memorial Hospital (India)	The hospital has a tumor biobank with approximately 75,000 patients' samples with clinical annotation. However, the follow-up data may be incomplete. The sequencing is done only if clinically indicated using the clinically relevant (targeted sequencing) panels
University of Utah (United States)	Received All of Us funding for genomic profiling of 1M patients in the United States. Recommends OMOP/OHDSI for standardized data submission; uses an EMR structure with 10K tables; aims to migrate to OMOP
UMF Iuliu Hatieganu Cluj-Napoca (Romania)	Holds genomic data, specifically transcriptomics, including microarray, RNAseq, spatial transcriptomics, and NGS (commercially available panels), for patients (700), clinical data, survival for patients with lung, thyroid, glioblastoma, colorectal, renal, prostate and other cancers
Vall d’Hebrón Institute of Oncology (VHIO; Spain)	Part of AACR GENIE; passed GDPR approvals for data sharing. Uses in-house tissue NGS panels (up to 500 genes) plus liquid biopsy assays (technology transfer Guardant360 CDx), both ISO-certified. From 2015 to 2024, 10,000 samples were profiled (2,000 only in 2024). Maintains shallow clinical curation of medical records (via REDCap) for the entire collection (tumor type, biopsy site, stage at timing of NGS, and survival status) and deep clinical curation (also via REDCap, PRISSMM model among others) for the subset of cases shared with GENIE BioPharma Collaborative (less than 200 cases, expanding gradually). Data modeled in OMOP format for improved interoperability. Has recently automated extraction of patient treatment exposure from pharmacy registries to enrich shallow clinical data in clinicogenomic studies. Uses internal instance of cBioPortal for cohort-level data exploration, including research-only panel sequencing
Vyas Cancer Research (VCR Park, India)	Has built a cloud-based platform to create and manage a cancer genomic registry and clinical trials. Enables screening, consenting, enrollment, and tracking of patients, whose samples are deidentified and tracked through an integrated LIMS and a clinical reporting system. Deidentified data sets (clinical/laboratory/longitudinal data) can be accessed/sorted/reanalyzed. Interfaces with EHRs with integrated conversion of handwritten notes to structured clinical data. The accumulation of patients in India is anticipated to begin from July 1, 2025 Purpose-built oncology-focused project management platform to manage multisite trials and enable access to patients Access to a retrospective repository of primarily FFPE blocks across most cancer types, with only basic annotation in most cases (∼200,000) and richer annotation in some cases (∼30,000)
Wake Forest University (United States)	Profiles most patients with third-party vendors (Tempus, Caris, etc) while raw data are available from some vendors, but not all. Tracks 18K cases in Southeastern United States, adding 8K more in the Midwest. Works to integrate demographic, treatment, and genomic information

Abbreviations: AACR, American Association for Cancer Research; AI, artificial intelligence; AE, adverse event; BH, Burjeel Holdings; Caris POA, Caris Life Sciences Precision Oncology Alliance; CNVs, copy-number variations; EHR, electronic health record; EMR, electronic medical record; FFPE, formalin-fixed paraffin-embedded; GDPR, General Data Protection Regulation; GENIE, Genomics Evidence Neoplasia Information Exchange; LIMS, laboratory information management system; MCGI, Maine Cancer Genomics Initiative; MGRB, Medical Genome Reference Bank; MSI, microsatellite instability; MTB, molecular tumor board; NCCCR, National Center for Cancer Care and Research; OHDSI, Observational Health Data Sciences and Informatics; OMOP, Observational Medical Outcomes Partnership; ORIEN, Oncology Research Information Exchange Network; PMT, Personalize My Treatment; QRBI, Qatar Biomedical Research Institute; SDOH, social determinants of health; SNVs, single nucleotide variations; TMB, tumor mutational burden; WIN, Worldwide Innovative Network.

A critical next step is not creation of additional isolated platforms, but alignment of existing efforts through a coordinated, consensus-driven approach. For many institutions, particularly in LMICs, the proliferation of partially overlapping frameworks creates practical barriers to participation, increases costs, and risks further widening global inequities.^22,23,64^ Key priorities include enabling interoperability across data models through standardized mappings and transition pathways,^65,66^ supporting context-specific use cases, and promoting sustainable and equitable funding structures that incentivize long-term participation.^67,68^ A neutral convening effort could help align stakeholders across academia, standards organizations, governments, and industry to reduce duplication and lower barriers to entry,^67,68^ and support context-specific use cases that reflect differing needs across resource settings.^64,69^

Global coordination does not require centralization. A federated model, where data remain locally governed but are accessible through shared standards and query frameworks, offers a feasible and equitable path forward.^68,70^ Such an approach must embed equity as a core design principle, ensuring that LMIC partners are meaningfully supported through capacity building, access to analytics, and participation in governance and authorship.^23,64,67^ In this context, WIN is well positioned to serve as a catalyst for alignment across initiatives.

## WIN's ROLE IN EXPANDING GLOBAL ACCESS AND DATA REPRESENTATION

This paper has outlined several obstacles to creating an effective open-access global database and highlighted potential solutions. WIN members collectively represent a demographically diverse patient population across 18 countries and five continents, giving the network strong potential to build one of the most inclusive cancer data sets worldwide. The WIN common database is conceived not as a standalone repository, but as a federated, globally inclusive framework designed to align existing data sets and initiatives, embed equity and LMIC participation, and leverage WIN's international clinical and molecular tumor board network. To achieve this, however, the consortium must prioritize expanding participation from underrepresented regions through targeted partnerships, resource support, and technical assistance. Many oncologists already generate real-world data but lack the analytical capacity to demonstrate its value to regulators, funders, or government agencies. A coordinated WIN collaboration can help bridge these gaps by promoting shared expertise and common methodologies.

The common database is intended not to duplicate existing standards, registries, or platforms, but to address a complementary gap at the intersection of global diversity, PO implementation, and clinical translation. Its distinct value lies in combining several features within one framework: explicit inclusion of underrepresented regions and LMICs; linkage of multimodal and longitudinal data to an international MTB and PO studies; and a federated, multistakeholder structure designed to align existing data sets and workflows across institutions rather than create another standalone repository. Table 5 summarizes key WIN differentiators relative to selected existing initiatives.

**TABLE 5:** Key Differentiators of the WIN Common Database

Initiative	Core Purpose	Primary Strength	Relative Limitation	Distinct Value of WIN
GA4GH	Develop global standards, policies, and frameworks to enable responsible genomic and health data sharing	Builds international consensus around interoperable standards, federated approaches, trust frameworks, and practical governance models	Builds international consensus around interoperable standards, federated approaches, trust frameworks, and practical governance models	Builds on such standards while linking multimodal and longitudinal cancer data to an international academic MTB, precision oncology studies, and real-world clinical translation
OHDSI/OMOP	Enable collaborative generation of reliable evidence from observational health care data through a standardized common data model	Standardization of diverse health care data into a common format that supports cross-site analytic interoperability	Primarily a data model and analytics framework rather than a dedicated global precision oncology implementation network	Uses OMOP-compatible harmonization within a broader precision oncology framework integrating molecular, clinical, and longitudinal outcome data across an international consortium
AACR Project GENIE	Accelerate precision oncology through a large international clinicogenomic registry	Large-scale aggregation of clinicogenomic data across cancer centers	More focused on clinicogenomic aggregation than on integrated MTB or study workflows, LMIC inclusion, and implementation across diverse resource settings	Emphasizes inclusion of underrepresented regions and LMICs, federated alignment across heterogeneous sites, and linkage to MTB-driven interpretation and precision oncology studies
cBioPortal networks	Provide an open-source platform for interactive visualization, exploration, and analysis of multidimensional cancer genomics data	Broad usability for genomic and clinical data interrogation	Primarily a portal and access layer rather than an operational framework for longitudinal, globally distributed, clinically embedded precision oncology data	Adds a federated, multistakeholder framework connecting data generation, harmonization, clinical interpretation, and translational use across institutions
National registries/country-specific databases	Support national-scale patient-level data capture	Strong country-specific depth, regulatory alignment, and local outcome tracking	Often limited by geography, variable cross-border interoperability, and underrepresentation of international diversity	Provides a globally inclusive framework designed to align data sets across countries, support cross-border interoperability, and expand representation from underrepresented populations

Abbreviations: AACR, American Association for Cancer Research; GA4GH, Global Alliance for Genomics and Health; GENIE, Genomics Evidence Neoplasia Information Exchange; LMICs, low- and middle-income countries; MTB, molecular tumor board; OHDSI, Observational Health Data Sciences and Informatics; OMOP, Observational Medical Outcomes Partnership; WIN, Worldwide Innovative Network.

The common database initiative provides WIN a unique opportunity to engage its diverse membership, from major academic centers to smaller community hospitals, in a unified data effort. By addressing challenges in data standardization and interoperability, WIN can help institutions with limited technical capacity adopt shared frameworks such as OMOP through common tools and expertise. This approach enables broader participation and positions WIN as a model for an inclusive, globally coordinated cancer data network.

WIN has conducted several investigator-initiated PO studies, including Investigational Device Exemption (IDE)^71^ and Investigational New Drug (IND)^72^ studies, and has shared its progress over 15 years through international symposia and publications.^71-86^ WIN's landmark international N-of-1 WINTHER trial^71^ opened a new era in PO by using transcriptomic data from tumor and normal analogous tissue of origin, in addition to genomic data, to guide personalized treatment selection based on a matching score algorithm.

WIN also convenes the only academic international MTB that provides multidisciplinary input on complex advanced cancer cases by incorporating patient multiomics and treatment factors into therapeutic recommendations. The consortium began publishing WIN-MTB cases, reflecting their educational and clinical value as PO becomes a more mainstream consideration alongside standard-of-care and clinical trials.^80^

In conclusion, establishing a global cancer treatment database would represent a transformative step for PO. Integrating longitudinal multimodal data, including genomic and other omics profiles, diagnostic imaging, clinical records, and trial outcomes, from diverse populations would substantially improve the precision and effectiveness of cancer care while accelerating scientific discovery across institutions and borders. Most importantly, such a resource would support more accurate and equitable treatment decisions for patients worldwide.

Building this database, however, requires overcoming significant challenges in data standardization, privacy regulation, and technical infrastructure. Broad adoption of international standards such as OMOP and OHDSI, flexible and transparent data-sharing agreements, and the use of advanced analytical technologies, including AI and distributed algorithms, will be essential to address these barriers.

Progress will depend on coordinated commitment from health care systems, industry, policymakers, and the global research community, while the patients' voice must be prominent. Investment in open data initiatives, robust infrastructure, and multidisciplinary expertise is essential to ensure that data can be shared responsibly, used effectively, and sustained over time. Finally, it is essential to advocate for responsible data sharing and to participate actively in discussions on data governance to protect patient interests while advancing PO.

WIN calls on partners across sectors, disciplines, and borders to join in realizing this vision. A unified global cancer database has the potential to transform cancer research and care, leading to an era in which PO becomes the standard for every patient.
